# Pancreatic Cancer: A Retrospective Study From the Najran Region of Saudi Arabia

**DOI:** 10.7759/cureus.65685

**Published:** 2024-07-29

**Authors:** Ahmed M Badheeb, Mohammad A Awad, Ali G Al Masad, Mohammed S Alyami, Mohammed A Fagihi, Mugahed Al Walani, Samer Alkarak, Hamad M Al Bahili, Abdallah Alatawi, Nadeem M Nagi, Ahmed R Madbouly, Abdullah Abu Bakar, Faisal Ahmed, Mohamed Badheeb

**Affiliations:** 1 Oncology, King Khalid Hospital-Oncology Center, Najran, SAU; 2 Medicine, Hadhramaut University, Mukalla, YEM; 3 Internal Medicine, King Khalid Hospital, Najran, SAU; 4 Gastroenterology and Hepatology, King Khalid Hospital, Najran, SAU; 5 Colorectal Surgery, King Khalid Hospital, Najran, SAU; 6 Surgical Oncology, King Khalid Hospital, Najran, SAU; 7 Gastroenterology and Advanced Endoscopy, King Khalid Hospital, Najran, SAU; 8 General Surgery, King Khalid Hospital, Najran, SAU; 9 Hepatobiliary Surgery, Prince Sultan Military Medical City, Riyadh, SAU; 10 Internal Medicine, University of Tabuk, Tabuk, SAU; 11 Oncology, King Khalid Hospital, Najran, SAU; 12 Ophthalmology, King Khalid Hospital, Najran, SAU; 13 Urology, Ibb University, Ibb, YEM; 14 Internal Medicine, Yale New Haven Health, Bridgeport Hospital, Bridgeport, USA

**Keywords:** saudi arabia, najran, pancreatic ductal adenocarcinoma, prognostic factors, mortality, chemotherapy, survival, gemcitabine, : pancreatic cancer

## Abstract

Background: Despite advances in treatment, pancreatic cancer frequently has a low survival rate due to its advanced-stage diagnosis. Treatment focuses on prolonging survival and maintaining quality of life. This study investigates the characteristics associated with survival in advanced pancreatic cancer patients treated at a single academic cancer center in Najran, Saudi Arabia.

Method: A retrospective chart review study covering the period January 1, 2015, and December 31, 2023, involved 80 adult patients with pathologically confirmed pancreatic cancer (ductal adenocarcinoma) at King Khalid Hospital in Najran, Saudi Arabia. Clinicopathological characteristics, therapy, response, and survival outcomes were all gathered and analyzed. The chi-squared test, Kaplan-Meier, and Cox proportional hazards method with hazard ratios (HR) and 95% confidence intervals (CI) were used for statistical analysis.

Result: The mean age was 65.7±14.1 years and 54 (67.5%) cases were male. The main symptom was abdominal pain (n=54, 67.5%), while jaundice was presented in 17 (21.2%) of cases. The baseline serum carbohydrate antigen 19-9 (CA 19-9) level varied among cases, with 35 (43.8%) having normal levels. The majority of cases (n=59, 73.8%) had distant metastases at the initial presentation, while 12 cases (15%) had localized disease (resectable), and 22 (27.5%) were locally advanced at the first presentation. The most commonly reported pathologic grade was poorly differentiated ductal adenocarcinoma in 39 (48.8%). FOLFIRINOX was used as first-line chemotherapy in 54 (67.5%) cases, while gemcitabine alone was used in 15 (18.8%) cases. First-line chemotherapy resulted in progressive disease in 30 (37.5%), stable disease in 30 (37.5%), and partial response in 14 (17.5%). With a mean follow‐up time of 14.8±8.6 months, 57 (71.2%) were dead, where the main cause of death was disease progression (n=51, 89.5%). The median overall survival was 13.5 months, with a 12-month survival rate of 56% and a 36-month survival rate of 17%. The median cancer-specific survival was 16 months (95% CI: 13-22 months). The 12-month median cancer-specific survival was 61% (95% CI: 51-73%), and the 36-month median cancer-specific survival was 19% (95% CI: 10-34%). In univariate analysis, initial metastasis presentation (HR: 35.46; 95% CI: 4.90-256.83, p<0.001), poor Eastern Cooperative Oncology Group Performance Status (ECOG-PS) (3-4) (HR: 2.34; 95% CI:1.34-4.09, p=0.003), and presence of multiple metastases (HR: 1.33; 95% CI: 1.09-1.62, p=0.004) were associated with worsened survival. Patients who received the first chemotherapy were associated with better survival (HR: 0.53; 95% CI: 0.29-0.98, p=0.043). Furthermore, the response rate in patients who received FOLFIRINOX was better than that of those who received gemcitabine alone, which was statistically significant (p=0.002).

Conclusion: Our study showed that initial metastatic presentation, poor ECOG-PS, and the occurrence of numerous metastases were all linked with poor survival of patients with pancreatic adenocarcinoma. Additionally, FOLFIRINOX as a first-line treatment showed better survival rates than gemcitabine alone. Raising awareness among healthcare providers on the alarming signs of pancreatic cancer and the introduction of personalized oncology might improve the outcome of this fatal malignancy.

## Introduction

Pancreatic cancer is an aggressive tumor with a poor prognosis, with a five-year overall survival rate of only 6% [1]. This is due to the advanced stage at diagnosis, making curative treatment unfeasible, and the lack of curative options [2]. It is the 14th most frequent malignancy and the seventh leading cause of cancer mortality worldwide [1]. Pancreatic cancer in Saudi Arabia has increased significantly in recent years, with new cases increasing from 131 in 2005 to 579 in 2022 [3,4]. The expected number of deaths from this cancer was 567 in 2022, according to the World Health Organization and International Agency for Research on Cancer (IARC) Global Cancer Observatory [4]. The five-year survival rate was the lowest among other cancers, ranking it eighth as a cause of cancer death in Saudi Arabia [3].

Despite minor increases in diagnoses, pancreatic cancer survival rates have not significantly improved. This is true despite recent advances in chemotherapeutic treatments, in part because the majority of patients may have asymptomatic locally advanced (40%) or metastatic disease (40%) at diagnosis [5]. More than 85% of pancreatic tumors are ductal epithelial adenocarcinomas, with less than 20% presenting with locally resectable disease [6]. Studies suggest that palliative treatment with multi-agent chemotherapy, such as a combination of 5-FU, oxaliplatin, and irinotecan (FOLFIRINOX) or gemcitabine with nab-paclitaxel, is the standard of care for metastatic pancreatic cancer, extending life expectancy by 6-8 months [7-9]. However, many trials have been undertaken for locally advanced pancreatic cancer; hence, these therapies are considered traditional first therapy [1].

According to studies, the initial recurrence in 76% of patients is systemic, implying that borderline resectable pancreatic cancer can be considered a systemic illness, even if there is no apparent metastatic disease on imaging [10,11]. Few local data describe the outcomes of pancreatic adenocarcinoma in Saudi Arabia, especially in the Najran region [12,13]. This report aims to investigate the treatment patterns with systemic regimens for pancreatic cancer and overall survival outcomes in real-world clinical practice.

## Materials and methods

Study design

A retrospective chart review study covered January 1, 2015, and December 31, 2023, involving 80 adult patients with pathologically confirmed pancreatic cancer (ductal adenocarcinoma) at King Khalid Hospital in Najran, Saudi Arabia. This study was approved by the Ethics Research Committees of King Khalid Hospital (KACST, KSA: H-I1-N-136) in compliance with the ethical standards outlined in the Declaration of Helsinki. Owing to the study's retrospective nature, written informed consent from the included patients was not required. Inclusion criteria include adult patients 18 years or older, with pathologically confirmed pancreatic cancer (ductal adenocarcinoma) at King Khalid Hospital in Najran, Saudi Arabia. In contrast, exclusion criteria included pathologically unconfirmed ductal adenocarcinoma pancreatic cancer, pediatric patients <18 years, neuroendocrine tumors, and those with incomplete medical records.

Initial evaluation, therapy, and follow-up

All patients had to undergo a biopsy to confirm their diagnosis of pancreatic cancer. A whole-body staging computed tomography (CT) scan and baseline values of carbohydrate antigen 19-9 (CA 19-9) and, in some patients, also carcinoembryonic antigen (CEA) were performed. A pancreatic magnetic resonance image (MRI) was requested only after the exclusion of distant metastases. A further imaging evaluation was ordered only for those going for resection. The tumor board determined resectability following the National Comprehensive Cancer Network (NCCN) guidelines [14]. At baseline, all patients' performance status was determined using the Eastern Cooperative Oncology Group Performance Status (ECOG-PS) (score range, 0-4, with lower values indicating greater performance). The number of metastatic locations was assessed using CT scan findings [15]. Cancer treatment was divided into four categories: single gemcitabine, gemcitabine plus nab-paclitaxel (GnP), FOLFIRINOX, or supportive care. Among the chemotherapeutic regimens, a single gemcitabine was selected as the reference. Specific comorbidities were mentioned as a total number of comorbidities (diabetes, hypertension, heart failure, renal failure, chronic respiratory failure, liver failure, history of thyroid disease) and weight loss (weight loss of ≥5% in the previous year). The NCCN guidelines define advanced pancreatic ductal adenocarcinoma as either locally advanced or metastasized. Locally advanced disease was defined as a solid tumor contact of 180° or more with the superior mesenteric artery or celiac axis or the inability to reconstruct the superior mesenteric vein or portal vein due to tumor involvement. Metastatic disease was defined as the presence of distant metastasis, including nonregional lymph node metastasis. The response to induction chemotherapy was classified as complete, progressive, no change (stable), and partial responses [16]. Overall survival is defined as the time from diagnosis to death, whereas progression-free survival (PFS) is the time between diagnosis and disease progression or death.

Collected data

The recorded data included age, gender, ECOG-PS score, pathologic grade and stage, metastasis number, CA 19-9 and CEA baseline, treatment modalities, response to treatment, and current status (survive or died).

Statistical analysis

We utilized the mean±standard deviation (SD) to represent the quantitative variables, and the frequency (percentage) was employed to describe the qualitative variables. Chi-squared tests were used to compare the characteristics of patients and tumors. Kaplan-Meier survival curves and Cox proportional hazards methods were applied for survival analyses. Factors included in survival analysis were age, gender, ECOG-PS, chemotherapy type, number of comorbidities (≤1 vs. >1), metastasis sites (mean±SD), jaundice, metastasis at initial presentation, ECOG-PS (0-2 vs. 3-4), pathology grade (well differentiated, moderately differentiated, and poorly differentiated), tumor presentation (localized, regional, and distant metastasis), and treatment characteristics. The relationship between pre-therapeutic variables and overall survival was reported as a hazard ratio (HR) with a 95% confidence interval (CI). A p-value less than 0.05 was deemed statistically significant. All the data were processed using IBM SPSS Statistics for Windows, Version 20.0 (Released 2011; IBM Corp., Armonk, New York, United States).

## Results

The mean age was 65.7±14.1 years (40-99 years), and 54 (67.5%) cases were male. Most cases, 26 (32.5%), were in ECOG-PS class 2. Comorbidities were presented in 49 (61.25%), with 22 (27.5%) having multiple comorbidities, 27 (33.8%) having one comorbidity, and 31 (38.8%) not reported any comorbidities. The commonly reported comorbidities were diabetes, hypertension, and cardiovascular disease in 29 (36.2%), 26 (32.5%), and nine (11.2%) of cases, respectively. The main symptoms were abdominal pain, anorexia, and weight loss in 54 (67.5%), 33 (41.2%), and 18 (22.5%) of cases, respectively, while jaundice was presented in 17 (21.2%) of cases. The baseline serum CA 19-9 level varied among cases, with 35 (43.8%) having normal levels, 18 (22.5%) having <500 U/mL, 11 (13.8%) having between 500 and 1000 U/mL, and four (5%) having highest levels (>40000 U/mL) (Table 1).

**Table 1:** Patients' clinical characteristics (n=80). ECOG-PS: Eastern Cooperative Oncology Group Performance Status; SD: standard deviation; CA 19-9: carbohydrate antigen 19-9; CEA: carcinoembryonic antigen; ng/Ml: nanograms per milliliter; U/mL: units per milliliter

Variables	Value
Age (years), mean±SD	65.7±14.1 (range 40.0-99.0)
Gender	
Male	54 (67.5%)
Female	26 (32.5%)
ECOG-PS	
0	6 (7.5%)
1	20 (25.0%)
2	26 (32.5%)
3	18 (22.5%)
4	10 (12.5%)
Comorbidities	
Diabetes	29 (36.2%)
Hypertension	26 (32.5%)
Cardiovascular disease	9 (11.2%)
Thyroid disease	6 (7.5%)
Pulmonary disease	2 (2.5%)
Chronic renal failure	2 (2.5%)
Chronic pancreatitis	2 (2.5%)
Autoimmune disease	1 (1.2%)
Patient's symptoms	
Anorexia	33 (41.2%)
Abdominal pain	54 (67.5%)
Weight loss	18 (22.5%)
Jaundice	17 (21.2%)
Serum CA 19-9 level	
Normal (<37 U/mL)	35 (43.8%)
<500 U/mL	18 (22.5%)
500-1000 U/mL	11 (13.8%)
1000-5000 U/mL	2 (2.5%)
5000-10000 U/mL	6 (7.5%)
10000-20000 U/mL	3 (3.8%)
20000-40000 U/mL	1 (1.2%)
>40000 ng/mL	4 (5.0%)
Serum CEA level	
Normal (<2.5 ng/mL)	39 (48.8%)
<200 ng/mL	37 (46.2%)
200-400 ng/mL	1 (1.2%)
>400 ng/mL	3 (3.8%)

The majority of cases (n=59, 73.8%) had distant metastases at the initial presentation, while 12 cases (15%) had localized disease (resectable), and 22 (27.5%) were locally advanced at the first presentation. The most commonly reported pathologic grade was poorly differentiated ductal adenocarcinoma in 39 (48.8%). The tumor was presented with distant metastasis in 46 (57.5%) cases and regional metastasis in 22 (27.5%) cases and was localized in 12 (15%) cases. The majority of metastasis was found in lymph nodes (n=36, 45%), followed by lung (36.2%), liver (n=15, 18.8%), and bone (n=14, 17.5%) (Table 2).

**Table 2:** Pathological characteristics (n=80).

Variables	Value
Differentiation (grade)	
Well differentiated	12 (15.0%)
Moderately differentiated	29 (36.2%)
Poorly differentiated	39 (48.8%)
Tumor presentation	
Localized	12 (15.0%)
Locally advanced	22 (27.5%)
Distant metastasis	46 (57.5%)
Initial metastasis presentation	59 (73.8%)
Metastasis location at presentation	
Lymph node metastasis	36 (45.0%)
Lung metastasis	29 (36.2%)
Bone metastasis	14 (17.5%)
Liver metastasis	15 (18.8%)
Other locations of metastasis	9 (11.2%)
Other gastrointestinal metastasis	7 (8.8%)
Renal metastasis	4 (5.0%)

Primary surgical resection was performed in 11 (13.8%) cases. FOLFIRINOX was used as first-line chemotherapy in 54 (67.5%) cases, while gemcitabine alone was used in 15 (18.8%) cases. First-line chemotherapy resulted in progressive disease in 30 (37.5%) cases, stable disease in 30 (37.5%) cases, partial response in 14 (17.5%) cases, and complete response in six (7.5%) cases. Second-line treatment includes palliative referral in 51 (63.8%) cases, gemcitabine alone in 24 (30%) cases, FOLFIRINOX in three (3.8%) cases, gemcitabine-nab-paclitaxel in one (1.2%) case, and post-chemotherapy surgical resection in one (1.2%) case. The outcome of second-line treatment includes progressive disease in 22 (73.3%) and stable disease in eight (26.7%) cases.

With a mean follow‐up time of 14.8±8.6 months (min: 4; max: 45 months), 57 (71.2%) were dead, and the majority of cases (n=51, 89.5%) were dead due to disease progression.

**Figure 1:**
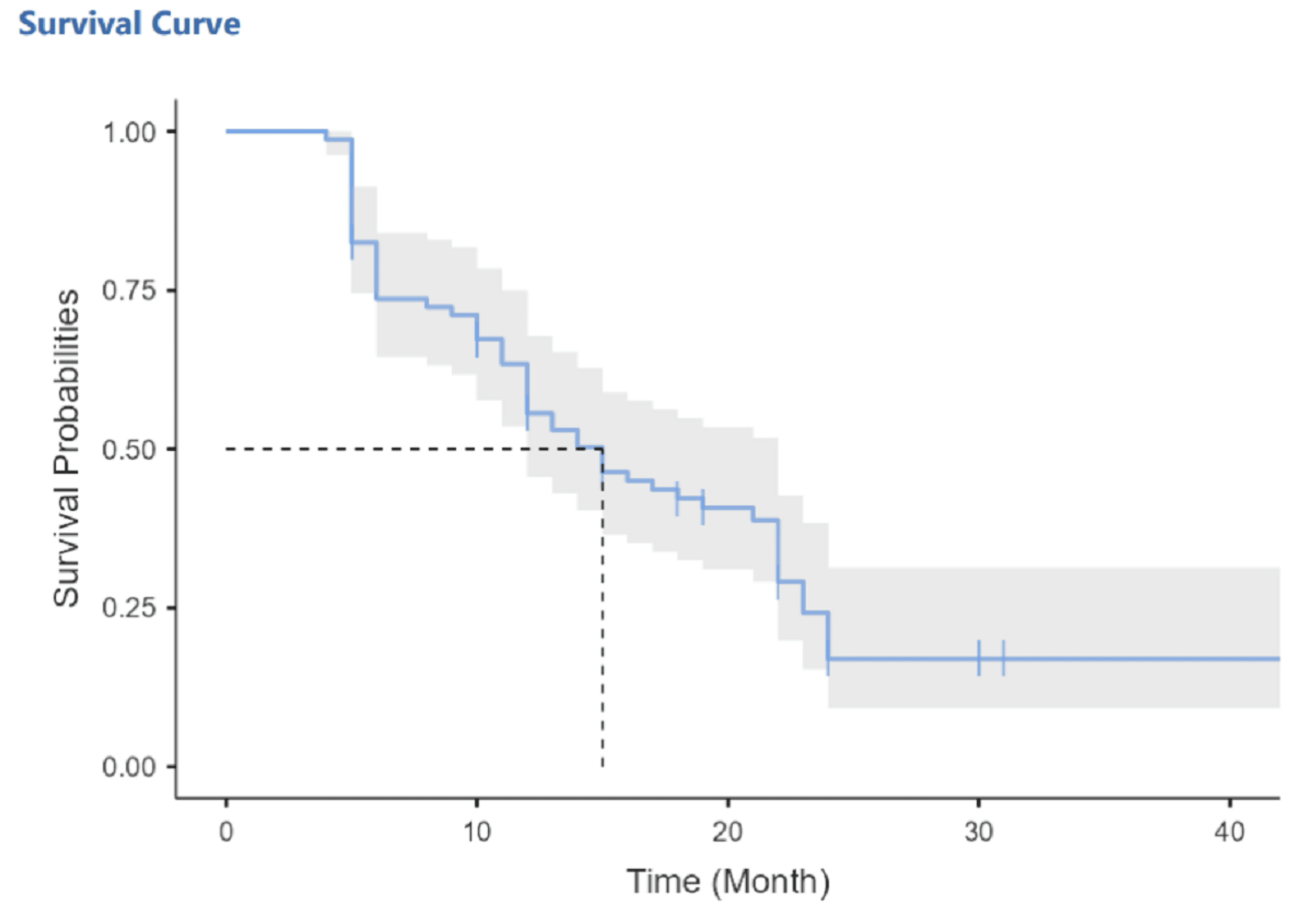
Kaplan-Meier survival curves for overall survival in pancreatic cancer cases.

The median survival time was 13.5 months (interquartile range (IQR): 6.0, 21.2 months; 95% CI: 12.00-22.00) (Table 3). The 12-month overall survival was 56% (95% CI: 46-68%), and the 36-month overall survival was 17% (95% CI: 9-31%) (Figure 1). The median cancer-specific survival was 16 months (95% CI: 13-22 months). The 12-month median cancer-specific survival was 61% (95% CI: 51-73%), and the 36-month median cancer-specific survival was 19% (95% CI: 10-34%) (Table 3).

**Table 3:** Treatment characteristics and survival outcome (n=80).

Variables	N (%)
Front-line therapy	
FOLFIRINOX	54 (67.5%)
Gemcitabine alone	15 (18.8%)
Surgical resection	11 (13.8%)
First-line outcome	
Progressive disease	30 (37.5%)
Stable disease	30 (37.5%)
Partial response	14 (17.5%)
Complete response (post-resection)	6 (7.5%)
Second-line treatment	
Palliative referral	51 (63.8%)
Gemcitabine alone	24 (30.0%)
FOLFIRINOX	3 (3.8%)
Gemcitabine-nab-paclitaxel	1 (1.2%)
Surgical resection	1 (1.2%)
Second-line outcome	
Progressive disease	22 (73.3%)
Stable disease	8 (26.7%)
Time survival (months), mean±SD	14.8±8.6 (range 4-45)
Time survival (months), median	13.5 (min: 4; max: 45)
Status	
Alive	23 (28.8%)
Dead	57 (71.2%)
Death causes	
Disease progression	51 (89.5%)
Sepsis	5 (8.8%)
Venous thromboembolism	1 (1.8%)

Factors associated with survival

**Figure 2:**
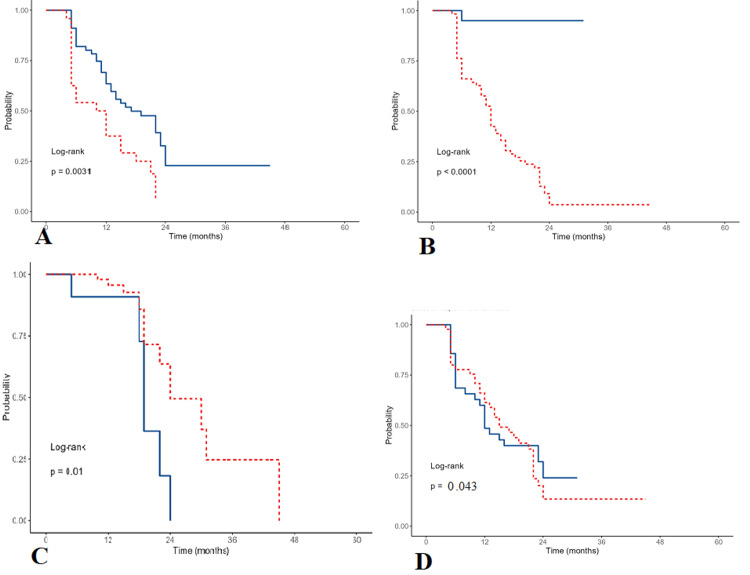
Survival plots divided by (A) number of metastases, (B) initial presentation with metastasis, (C) Eastern Cooperative Oncology Group Performance Status, and (D) treatment with different chemotherapy types (present: red dashed line) (absent: blue continuous line).

In univariate analysis, initial metastasis presentation (HR: 35.46; 95% CI: 4.90-256.83, p<0.001), poor ECOG-PS (3-4) (HR: 2.34; 95% CI:1.34-4.09, p=0.003), and presence of multiple metastases (HR: 1.33; 95% CI: 1.09-1.62, p=0.004) were associated with worsen survival and were statistically significant (Figure 2). Other factors such as multiple comorbidities (HR: 1.01; 95% CI: 0.48-2.17), gender (HR: 1.18; 95% CI: 0.68-2.05), high serum CA 19-9 level (HR: 1.09; 95% CI: 0.64-1.84), higher serum CEA level (HR: 1.27; 95% CI: 0.75-2.15), and poorly differentiated tumor grade (OR: 1.81; 95% CI: 0.80-4.09) were associated with worsen survival but were not statistically significant (all p-values >0.05) (Table 4).

**Table 4:** Factors associated with mortality in univariate analysis. ECOG-PS: Eastern Cooperative Oncology Group Performance Status; SD: standard deviation; CA 19-9: carbohydrate antigen 19-9; CI: confidence interval; HR: hazard ratio

Variables	Subgroup	Total	HR (95% CI)	P-value
Gender	Male	54 (67.5)	Reference group	0.556
	Female	26 (32.5)	1.18 (0.68-2.05)	
Initial metastasis presentation	No	21 (26.2)	Reference group	<0.001
	Yes	59 (73.8)	35.46 (4.90-256.83)	
Diabetes mellitus	No	51 (63.8)	Reference group	0.938
	Yes	29 (36.2)	0.98 (0.57-1.68)	
Jaundice	No	63 (78.8)	Reference group	0.856
	Yes	17 (21.2)	1.07 (0.54-2.11)	
Surgical resection	No	69 (86.2)	Reference group	0.343
	Yes	11 (13.8)	0.64 (0.26-1.61)	
First chemotherapy use	No	15 (18.8)	Reference group	0.043
	Yes	65 (81.2)	0.53 (0.29-0.98)	
Second chemotherapy use	Only palliative care	29 (36.2)	Reference group	0.272
	Yes	51 (63.8)	0.74 (0.44-1.26)	
Serum CA 19-9 level	Normal (<37 U/mL)	35 (43.8)	Reference group	0.761
	High	45 (56.2)	1.09 (0.64-1.84)	
Serum CEA level	Normal (<2.5 ng/mL)	39 (48.8)	Reference group	0.378
	High	41 (51.2)	1.27 (0.75-2.15)	
Tumor grade	Well differentiated	12 (15.0)	Reference group	0.526
	Moderately differentiated	29 (36.2)	0.74 (0.30-1.86)	
	Poorly differentiated	39 (48.8)	1.81 (0.80-4.09)	0.153
Comorbidity number	No comorbidity	31 (38.8)	Reference group	0.191
	One comorbidity	27 (33.8)	1.50 (0.82-2.74)	
	Multiple comorbidities	22 (27.5)	1.01 (0.48-2.17)	0.970
ECOG-PS	Low (0-2)	56 (70.0)	Reference group	0.003
	High (3-4)	24 (30.0)	2.34 (1.34-4.09)	
Age (year)	Mean±SD	65.7±14.1	0.99 (0.97-1.00)	0.139
Multiple metastasis	Mean±SD	1.4±1.1	1.33 (1.09-1.62)	0.004

Patients who received the first-line chemotherapy were associated with better survival (HR: 0.53; 95% CI: 0.29-0.98, p=0.043) and were statistically significant (Figure 2D).

The statistically significant factors associated with improved survival were well-differentiated tumor grade, no metastasis at presentation, low metastasis numbers, and FOLFIRINOX as the first chemotherapy (Table 5).

**Table 5:** Comparison between survived and dead patients. ECOG-PS: Eastern Cooperative Oncology Group Performance Status; SD: standard deviation; CA 19-9: carbohydrate antigen 19-9; CEA: carcinoembryonic antigen

Variables	Subgroup	Alive	Dead	P-value
Age (year)	Mean±SD	66.7±16.1	65.3±13.4	0.691
Gender	Male	16 (69.6)	38 (66.7)	1.000
	Female	7 (30.4)	19 (33.3)	
ECOG-PS	Low	20 (87.0)	36 (63.2)	0.036
	High	3 (13.0)	21 (36.8)	
Tumor grade	Well differentiated	5 (21.7)	7 (12.3)	<0.001
	Moderately differentiated	15 (65.2)	14 (24.6)	
	Poorly differentiated	3 (13.0)	36 (63.2)	
Comorbidity	No comorbidity	9 (39.1)	22 (38.6)	0.553
	One comorbidity	6 (26.1)	21 (36.8)	
	Multiple comorbidity	8 (34.8)	14 (24.6)	
Metastasis at presentation	No	20 (87.0)	1 (1.8)	<0.001
	Yes	3 (13.0)	56 (98.2)	
Metastasis number	Mean±SD	0.6±0.9	1.8±1.0	<0.001
Serum CEA level	Normal	14 (60.9)	25 (43.9)	0.258
	High	9 (39.1)	32 (56.1)	
Serum CA 19-9 level	Normal	12 (52.2)	23 (40.4)	0.474
	High	11 (47.8)	34 (59.6)	
Tumor presentation	Localized	4 (17.4)	8 (14.0)	0.826
	Regional	7 (30.4)	15 (26.3)	
	Distant metastasis	12 (52.2)	34 (59.6)	
Survival time (months)	Mean±SD	21.7±9.3	11.9±6.6	<0.001
First chemotherapy use	Resection	6 (26.1)	5 (8.8)	0.028
	FOLFIRINOX	16 (69.6)	38 (66.7)	
	Gemcitabine alone	1 (4.3)	14 (24.6)	
Second chemotherapy use	FOLFIRINOX	1 (4.3)	2 (3.5)	0.072
	Gemcitabine-nab-paclitaxel	1 (4.3)	0 (0.0)	
	Gemcitabine alone	3 (13.0)	21 (36.8)	
	Resection	1 (4.3)	0 (0.0)	
	Palliative referral	17 (73.9)	34 (59.6)	

The responses in patients who received FOLFIRINOX as a first treatment were better than gemcitabine alone and were statistically significant (p=0.002) (Table 6).

**Table 6:** Response rate in FOLFIRINOX and gemcitabine alone used as the first chemotherapy treatment in pancreatic cancer.

Variables	Subgroups	Total	Surgical resection	FOLFIRINOX	Gemcitabine alone	P-value
The outcome	Progressive disease	30 (37.5)	2 (18.2)	22 (40.7)	6 (40.0)	0.002
	Complete response (post-resection)	6 (7.5)	4 (36.4)	1 (1.9)	1 (6.7)	
	Stable disease	30 (37.5)	5 (45.5)	22 (40.7)	3 (20.0)	
	Partial response	14 (17.5)	0 (0.0)	9 (16.7)	5 (33.3)	

## Discussion

The survival rate for metastatic pancreatic cancer is relatively low. However, few studies have looked at the survival rate and therapeutic effect concerning the number and location of metastases and the initial treatment employed [17]. This study investigated the characteristics associated with survival in pancreatic cancer patients treated at a single cancer center in Najran, Saudi Arabia. The study's primary findings were that initial metastatic presentation, poor ECOG-PS, and numerous metastases were all linked with poor survival. Additionally, patients who received FOLFIRINOX as their first treatment had improved survival rates than those who got gemcitabine alone.

This study's mean age was 65.7±14.1 years, and most cases were male (67.5%). Our results were in line with previous reports from Saudi Arabia [2,3]. For example, Elwali et al. mentioned that over 85% of pancreatic cancer cases were diagnosed in individuals over 50, with 60-64 years old being the largest age group [3]. However, our patients were younger than other reports from Western countries, such as the United States, who reported the mean age for pancreatic cancer as 72 years [18]. These differences may be attributed to the country's younger population or cancer's unique biology. Furthermore, our study confirms previous reports on male-predominant pancreatic cancer [2,3]. Still, the reasons behind this higher incidence are not fully understood, possibly due to women's reduced sensitivity to malignant tumors. A 15-study review found reproductive factors don't correlate with pancreatic cancer in women, suggesting environmental or genetic factors may explain male predominance [19].

Our study discovered that 61.25% of patients had comorbidities, notably diabetes and hypertension, which is comparable with previous Saudi Arabian studies such as AlGhamdi et al., who revealed that nearly half of the patients had these conditions [20].

Our study revealed that abdominal pain and anorexia were the most common symptoms in 67.5% and 41.2%, respectively, which is comparable with previous studies [19,20], while only 21.2% presented with jaundice, which may prolong the interval between the onset of symptoms and the treatment as reported in the literature [20]. The delay in the presentation may result in a more advanced stage of diagnosis.

Most of our patients were in the advanced stage; 46 (57.5%) were presented with distant metastasis, and 22 (27.5%) had regional metastasis. At the same time, only 12 (15%) had localized pancreatic cancer at presentation. Furthermore, the univariate analysis showed that initial metastasis presentation (HR: 35.46) was associated with worsened survival in pancreatic cancer. Our result aligned with previous reports such as AlGhamdi et al., Alalawi et al., and Bilici [20-22].

Pancreatic cancer can spread through lymphogenic, hematogenous, and perineural pathways. The liver is the most common metastatic location, followed by the lymph nodes, lung, and peritoneum. Other less common sites include the kidney, adrenal gland, bone, spleen, gallbladder, omentum, and brain [17]. However, metastatic patterns in our report varied, with lymph node metastasis being the most common site. These conflicting findings may be attributed to small sample sizes and advanced stages in our cases. More prospective and randomized clinical trials with large numbers are necessary to validate our findings.

Previous studies have found a correlation between distant metastases and outcomes in pancreatic cancer [17,23,24]. In this study, the number of metastatic sites was found to be an independent prognostic factor for worsening survival in patients with metastatic pancreatic cancer. In another study, Wu et al. found a similar correlation, and patients with one- or two-site metastasis had more prolonged overall survival with combination therapy compared to monotherapy or no chemotherapy. However, patients with more than two metastatic sites had no superior overall survival with combination therapy or monotherapy [17]. In the present study, we also discovered that high ECOG-PS (PS 3-4) was substantially linked with poorer survival in patients with all stages of pancreatic cancer (HR: 2.34). This finding was similar to previous reports such as Sezgin et al. and Tas et al. [25,26].

Comorbidities such as diabetes mellitus and hypertension and their association with worsened survival were reported with variety in the literature; however, there is insufficient knowledge base to conclude this topic [27]. Conti et al. found that medication use for diabetes mellitus was a protective factor for survival in metastatic pancreatic cancer [15]. Napoli et al. investigated the factors predicting survival in patients with locally advanced pancreatic cancer undergoing pancreatectomy with arterial resection. They identified eight prognostic factors, four of which had a negative impact and four a protective value. The negative prognostic factors were platelet count, CA 15.3 level, CA 125 level, and neutrophil-to-lymphocyte ratio. The protective factors were metabolic deterioration of diabetes, lymphocyte-to-monocyte ratio, platelet-to-lymphocyte ratio, and FOLFIRINOX-based neoadjuvant chemotherapy [28]. In another study, AlGhamdi et al. revealed that age and tumor characteristics significantly impact survival in patients with advanced pancreatic cancer. Age, grade, abdominal lymph node, and liver metastasis were associated with poorer survival. High tumor markers CA 19-9 and CEA levels were also linked to poorer survival [20]. Bahardoust et al. found that age ≥60 years, weight loss, poor differentiation, tumor size >2.5 cm, metastasis presence, more than two involved lymph nodes, and lymph node ratio <0.2 were associated with worsened outcomes. In contrast, adjuvant therapy with surgery and chemotherapy was associated with better outcomes [29]. In this study, factors such as multiple comorbidities, gender, high serum CA 19-9 level, high serum CEA level, and poorly differentiated tumor grade were associated with mortality. Still, they were not statistically significant (all p-values >0.05) which may be due to the low sample size (80 cases). Overall, predicting outcomes for pancreatic cancer requires the consideration of multiple confounding factors, including clinical factors, pathological factors, laboratory and molecular factors, and the treatment used [22]. Further prospective studies are imperative to enhance our understanding of this cancer outcome.

FOLFIRINOX and gemcitabine plus nab-paclitaxel are the favored first-line chemotherapeutic regimens for patients with advanced ductal adenocarcinoma [30,31]. Our study revealed that first-line chemotherapy was associated with increased patient survival, which was consistent with prior findings in Saudi Arabia and other reports such as Bahardoust et al. from Iran, where patients who did not undergo surgery or chemotherapy had lower survival [20,29]. Furthermore, we found that using FOLFIRINOX as the first chemotherapy was more effective than gemcitabine alone and associated with better survival. In another cohort study, FOLFIRINOX was linked to higher partial response and pancreatectomy rates in patients with localized pancreatic adenocarcinoma compared to gemcitabine-nab-paclitaxel, but overall survival was similar [32]. Two randomized trials found that FOLFIRINOX and gemcitabine-nab-paclitaxel regimens prolong survival compared to gemcitabine. Patients receiving FOLFIRINOX had longer median overall survival duration and higher radiographic response rates but higher associated adverse events [33,34]. In another report, overall survival favored nab-paclitaxel+gemcitabine versus gemcitabine (41.8 vs. 37.7 months) in the five-year follow-up analysis [35]. These regimens are increasingly used as first-line treatment for localized disease, often with preoperative intent [32]. The recently reported results showed that adjuvant FOLFIRINOX, but not gemcitabine plus nab-paclitaxel (GA), definitively prolonged disease-free survival following pancreatectomy relative to gemcitabine that may have reinforced the belief that FOLFIRINOX may be more effective in patients with localized pancreatic ductal adenocarcinoma [35,36]. Costa et al. found a median overall survival of 6.7 months (gemcitabine alone) to 13.8 months (FOLFIRINOX) in metastatic pancreatic tumors [37]. We confirmed that the gemcitabine alone regimen is less effective in individuals with metastatic pancreatic tumors. However, because our study was conducted retrospectively, the dose-intensity-related feasibility of each regimen remained unknown. Additionally, resistance to existing chemotherapies such as gemcitabine is limiting viable treatment options, prompting the development of new treatments. Synthetic lethality and immunotherapy have shown promising results. Monoclonal antibodies are being used in clinical trials alongside chemotherapy and immune checkpoint inhibitors. By understanding the underlying pathogenic mechanisms and refining therapeutic approaches, breakthroughs are expected to pave the way for more effective treatments for this challenging disease [38].

Pancreatic cancer recurrence is common in patients undergoing curative surgery. Early metastasis of cancer cells to other organs, such as the liver, may explain the high recurrence rate after resection [32]. Our study found that primary surgical resection was performed in 13.8% of cases, and first-line chemotherapy was used in 54.5%. The outcome of first-line chemotherapy was progressive disease in 30 (37.5%), stable disease in 30 (37.5%), partial response in 14 (17.5%), and complete response (post-resection) in six (7.5%) cases. Adjuvant chemotherapy has been shown to improve survival rates, but 30-40% of patients are unable to receive it due to postoperative complications [1]. Delays and dose modifications are shared in those receiving adjuvant treatment. Additionally, 50% of patients treated with surgical resection and adjuvant chemotherapy will still relapse within two years [38].

In this study, pancreatic cancer has a mortality rate of 71.2%, with a median overall survival of 13.5 months and a median cancer-specific survival of 16 months, with a 12-month survival rate of 56% and a 36-month survival rate of 17%. In another report by Kent et al., the median overall survival was 22 months, with an anticipated survival rate of 70% (one year), 39.5% (two years), and 24% (three years) [39]. In the Bahardoust et al. study, the one- and three‐year survival rates were estimated to be 56.6% and 17.6%, respectively [29]. Survival rates for pancreatic cancer may vary due to undiscovered tumor biology aspects. Standard staging is based on the TNM approach, although a recent study underlines the importance of tumor grade in prognosis. Conditional survival enables the revision of survival estimates over time despite a lack of knowledge about individual tumor biology characteristics [40].

Study limitations

This study has been limited by its retrospective methodology and small sample size, which assessed the electronic medical records of cancer patients who attended the King Khalid Hospital in Najran, Saudi Arabia. Moreover, because this is a single-center study, it cannot exclude possible selection biases. A retrospective document review for patients' ultimate diagnosis may be unrelated to their principal complaint, which needed to be more comprehensive and sensitive to cancer and treatment-related tools. Further prospective studies are required to help guide the chemotherapy treatment utilizing a prospective registry of consecutive cases in their pharmacovigilance series.

## Conclusions

Our study showed that initial metastatic presentation, poor ECOG-PS, and the occurrence of numerous metastases were all linked with poor survival of patients with localized pancreatic adenocarcinoma. Additionally, FOLFIRINOX as a first-line treatment showed better survival rates than gemcitabine alone. Raising awareness among healthcare providers on the alarming signs of pancreatic cancer and introducing personalized oncology might improve the outcome of this fatal malignancy.
